# Supplementary material for: Kinome-Wide Virtual Screening by Multi-Task Deep Learning
Source: Int J Mol Sci. 2024 Feb 22;25(5):2538. doi: 10.3390/ijms25052538 (PMC10932040; doi:10.3390/ijms25052538)
Supplement: Supplementary file 1 [file ijms-25-02538-s001.zip › ijms-2800403-supplementary/Document S1/Hu_MTDNN_Supporting_Figures_Feb04_2024.pdf]

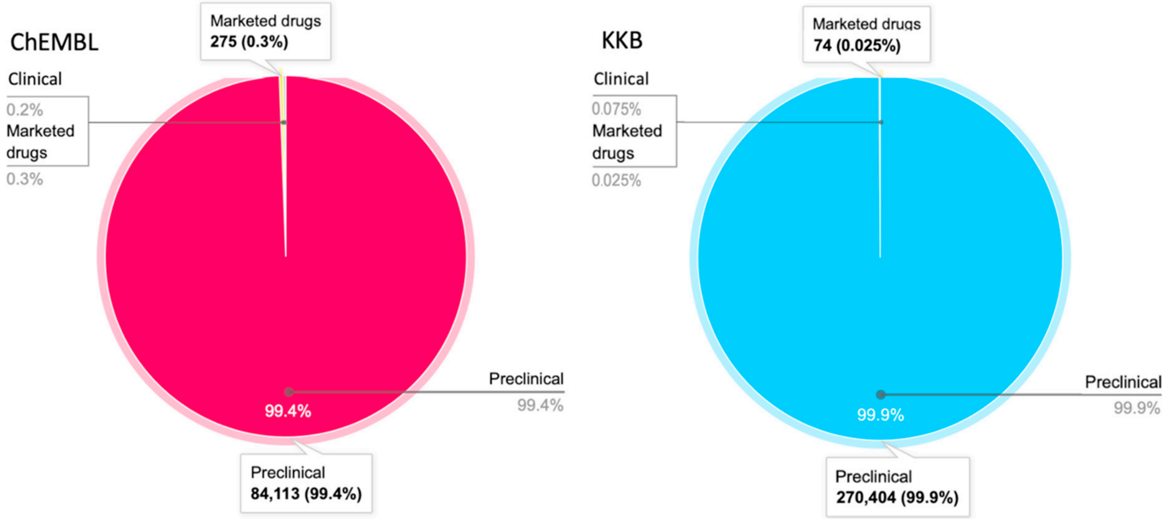


**Figure S1.** Percentage of compound developmental status from ChEMBL and KKB datasets.

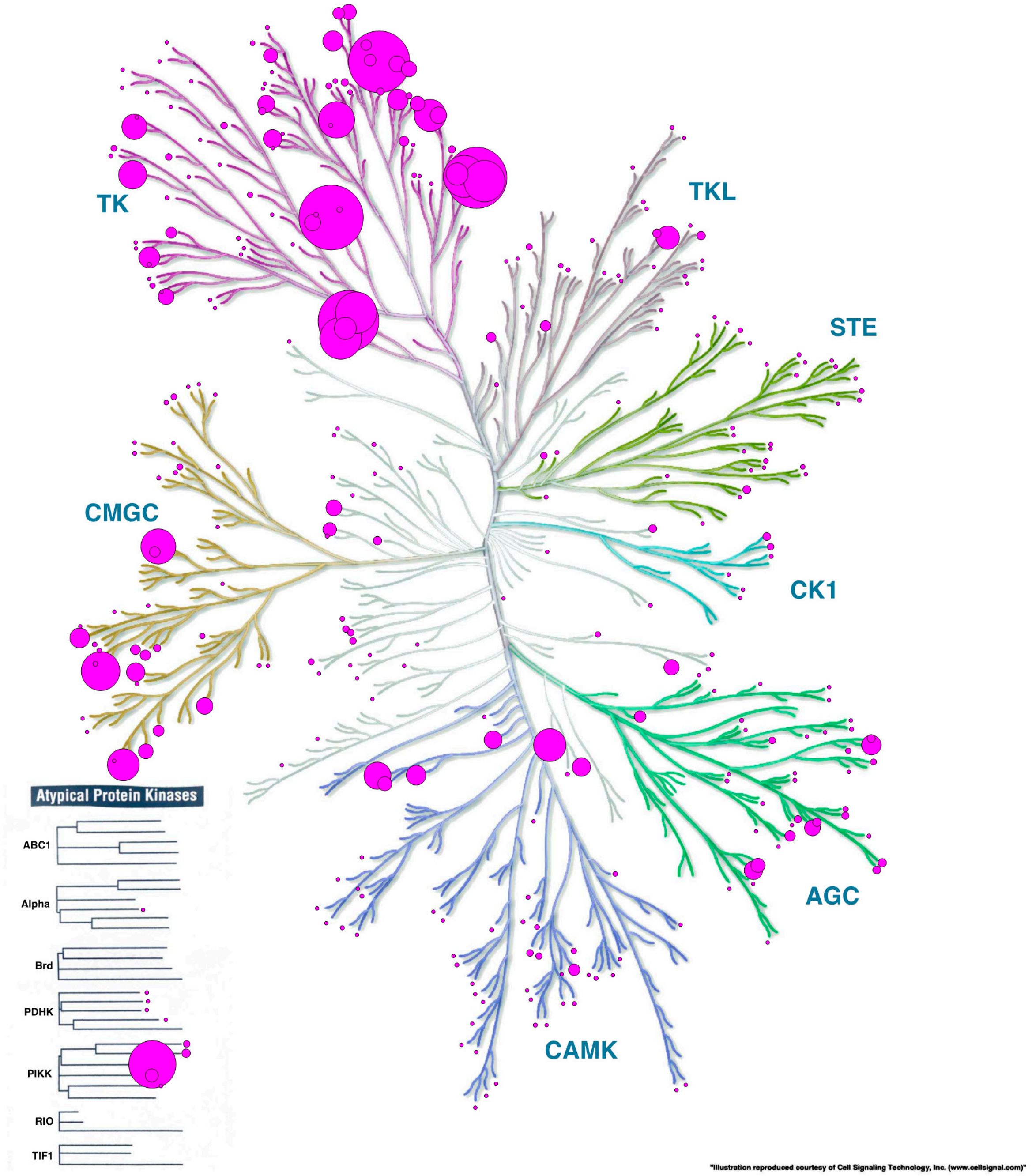


**Figure S2.** Distribution of kinase bioactivity data from our aggregated datasets.

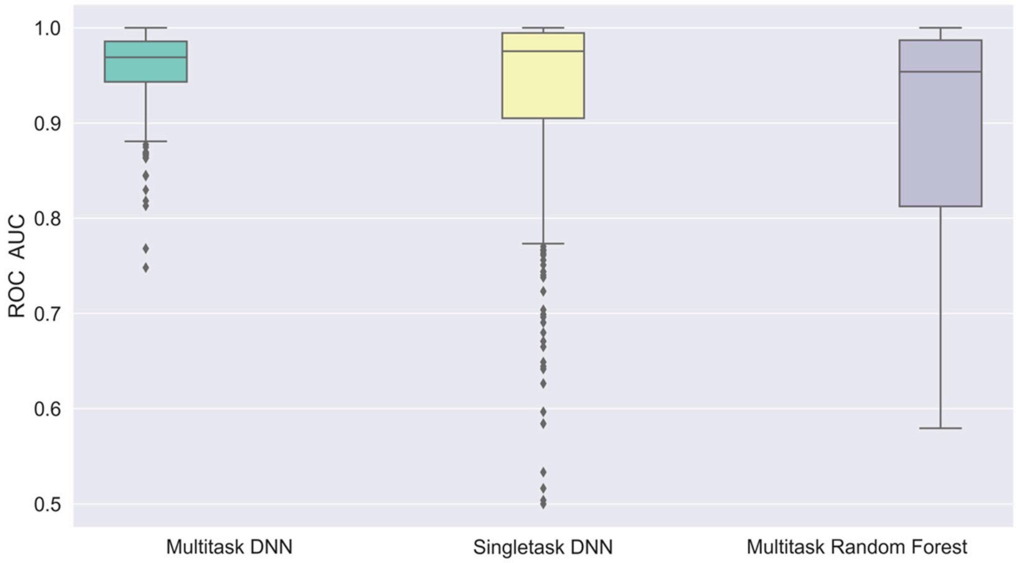


**Figure S3.** ROC AUC comparison between known active-presumed inactive (KAPI) Multitask DNN, Singletask DNN, and Multitask Random Forest models using random stratified 5-fold cross-validation.

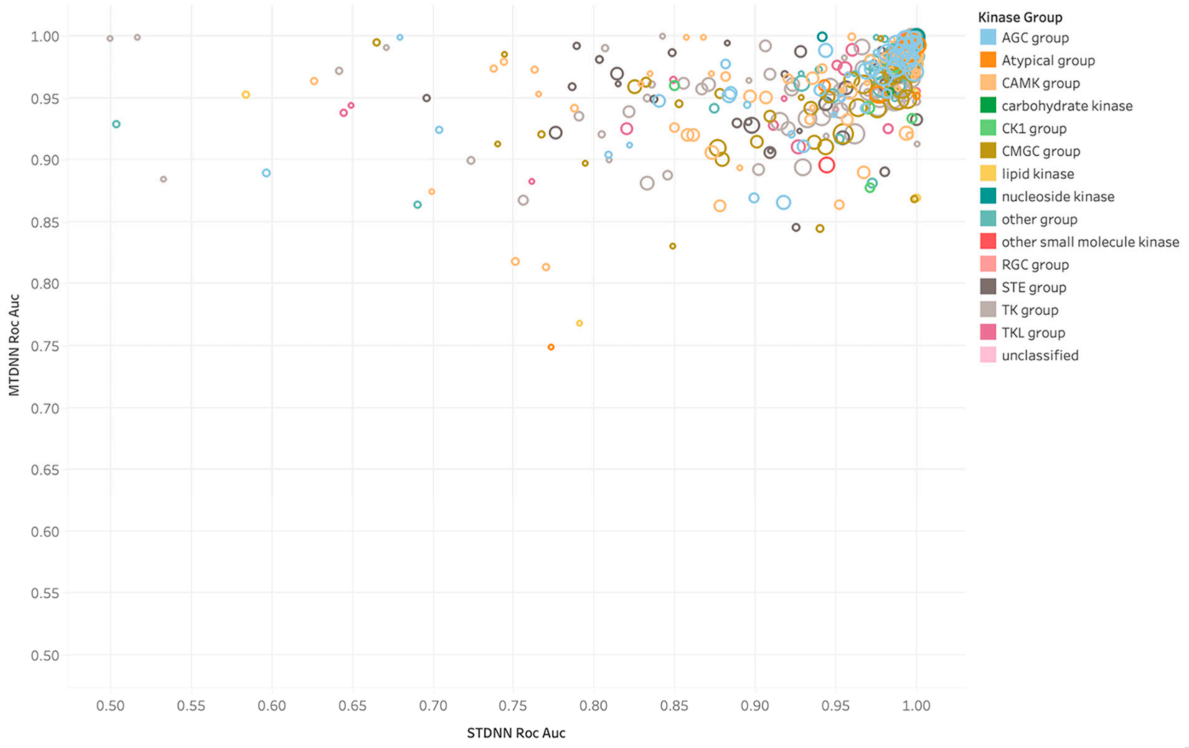


**Figure S4.** Scatterplot of known active-presumed inactive (KAPI) Multitask DNN and Singletask DNN models featuring different colored labels for kinase groups, with smaller circles representing lower datapoints, and bigger circles indicating higher datapoints.

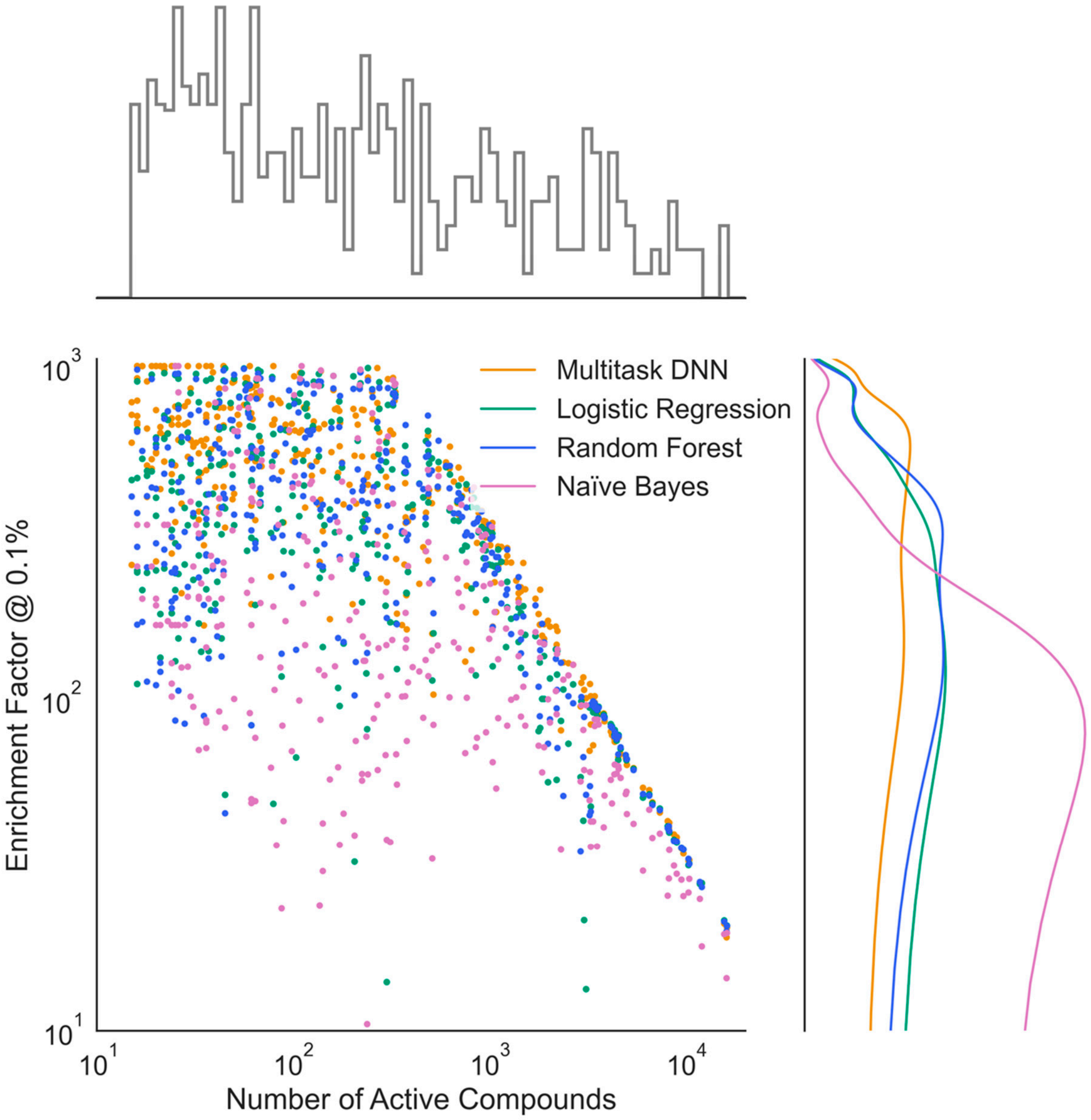


**Figure S5.** Enrichment factor as a function of active compounds at 0.1% for single and multitask 342 kinases known active-presumed inactive (KA-PI) classifiers. Single-task logistic regression, random forest, naïve Bayes, and multitask DNN modeling methods are shown along with the distribution of the enrichment of active compounds at 0.1% of the dataset.

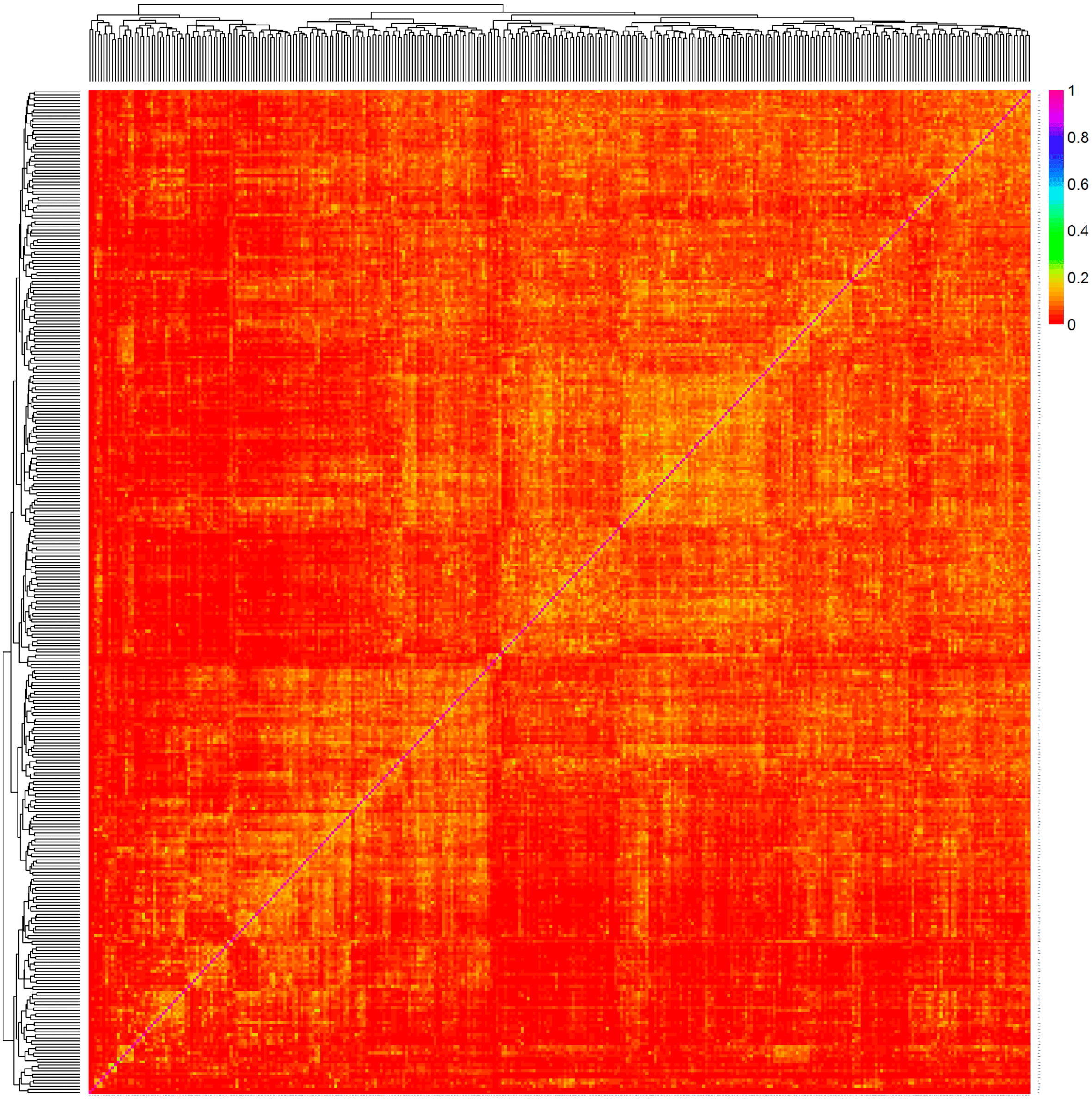


**Figure S6.** Pairwise distance similarity matrix of kinase compound cluster centers. The clustering shows diverse molecules with low similarities between each other. This method was used to split compounds by scaffold to cross-validate different machine learning methods.

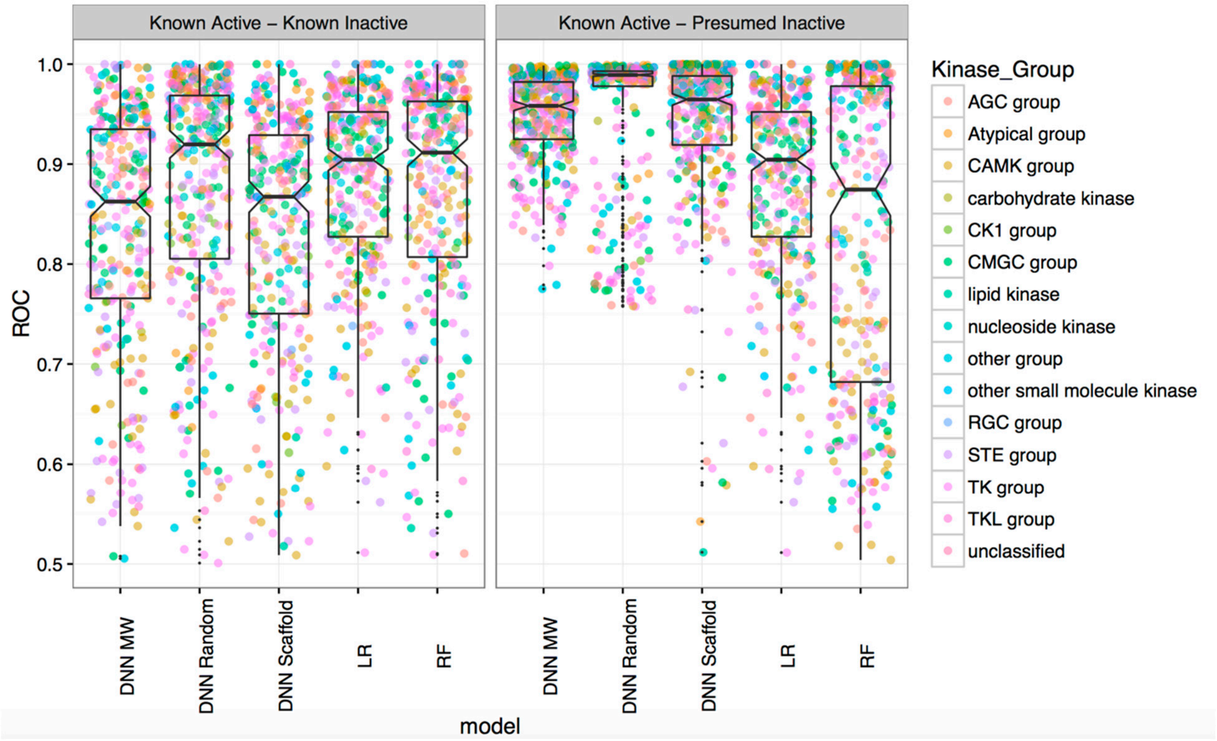


**Figure S7.** Differences in ROC score for kinase learning methods. Boxplot with ROC score data distribution for all 342 kinase tasks. Each box represents a specific model using either the KA-KI or KA-PI datasets. Models prefixed by DNN are the multitask deep neural network models, which were cross-validated by molecular weight (MW), scaffold, or random splits (see methods). LR – Logistic Regression, RF – Random Forest. Points are colored by their kinase group membership.

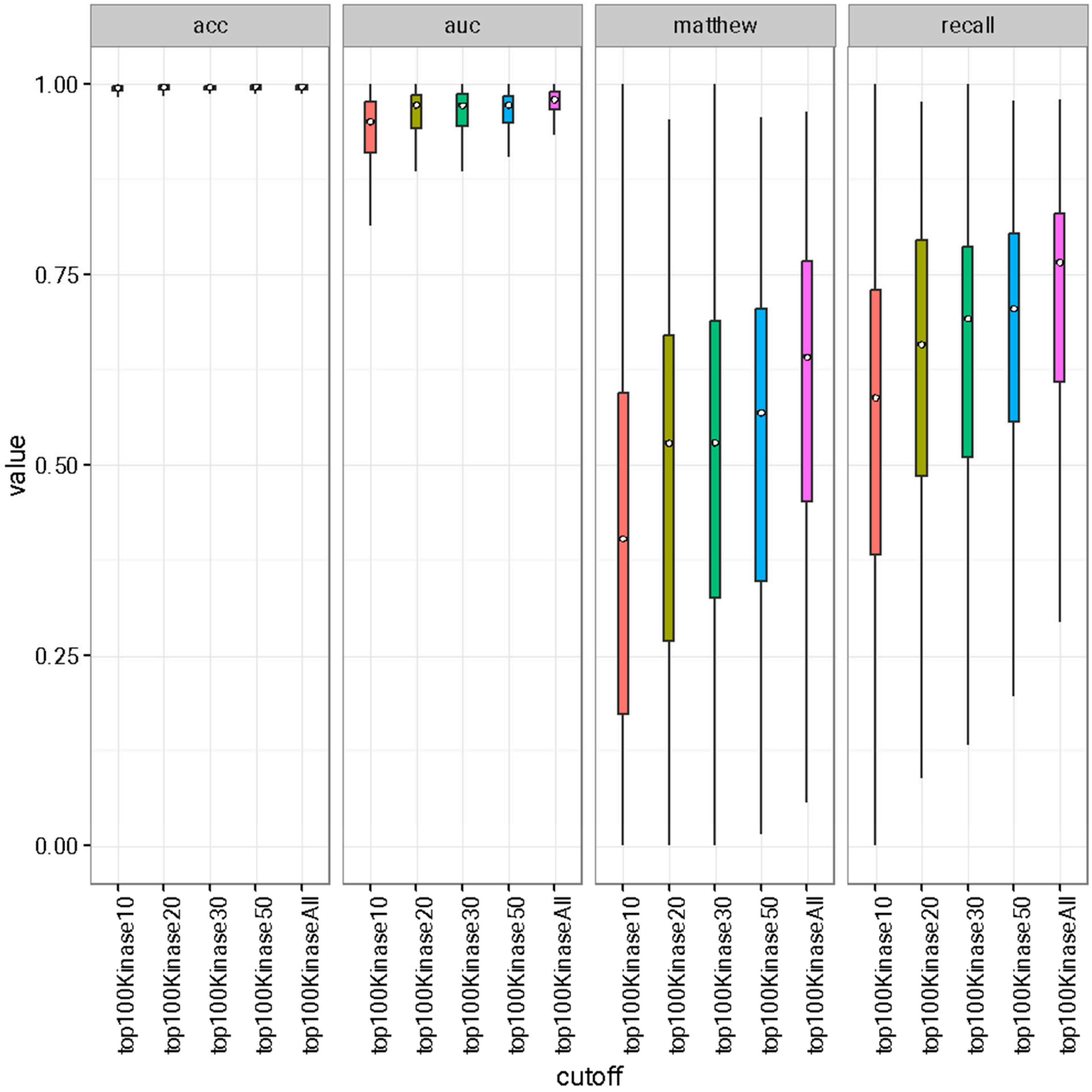


**Figure S8.** Data dependence of multitask deep neural networks. MTDNN increases performance as the amount of training data increases. This is in contrast to single-task machine learning approaches, which plateau more quickly or even decrease in performance as data increases (not shown).

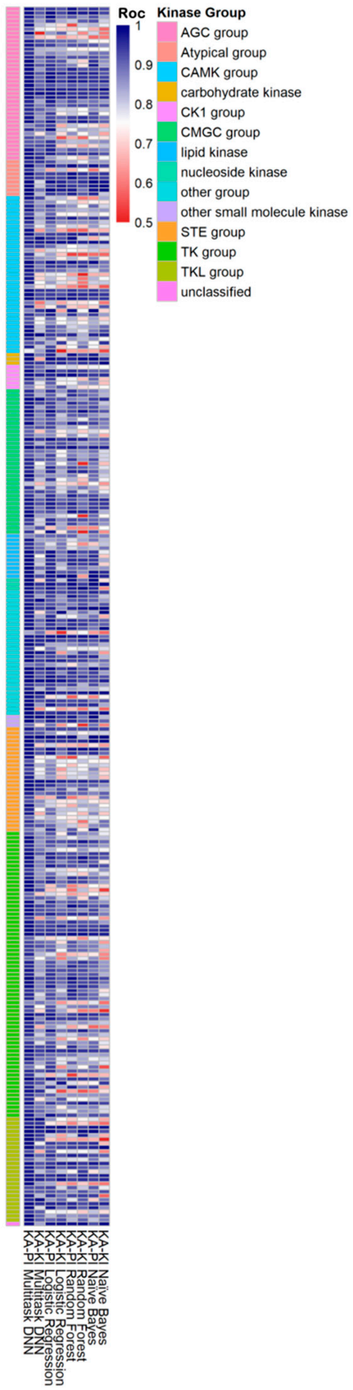


**Figure S9.** ROC scores for different modeling methods by kinase groups. Heatmap representation displaying all results from single- and multi-task modeling methods. For each method, the top panel shows results for the known active–known inactive (KA-KI) datasets and the bottom panel shows results for the known active–presumed inactive (KA-PI) datasets.

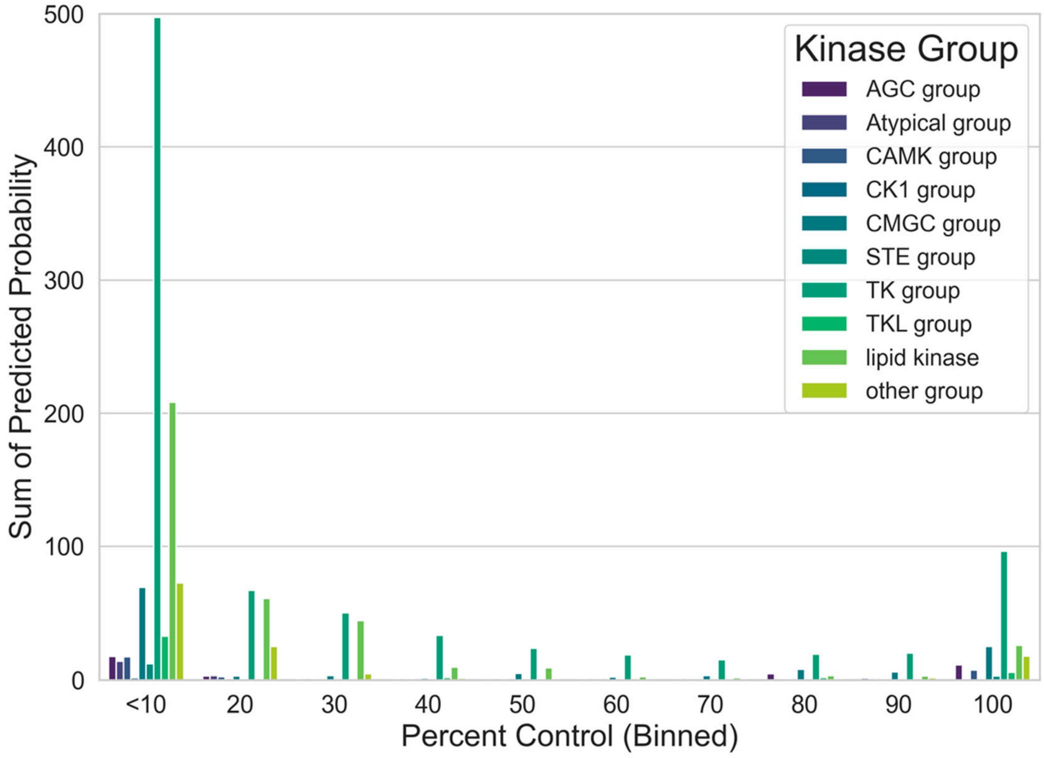


**Figure S10.** Summed predicted probabilities of activity (by kinase category) for mapped LINCS KINOMEScan profiling compound-target combinations, binned by percent control (lower percent control corresponds to higher inhibitory activity). The sum of probabilities appears high for the inactive compounds (100% control), because most compounds are inactive.

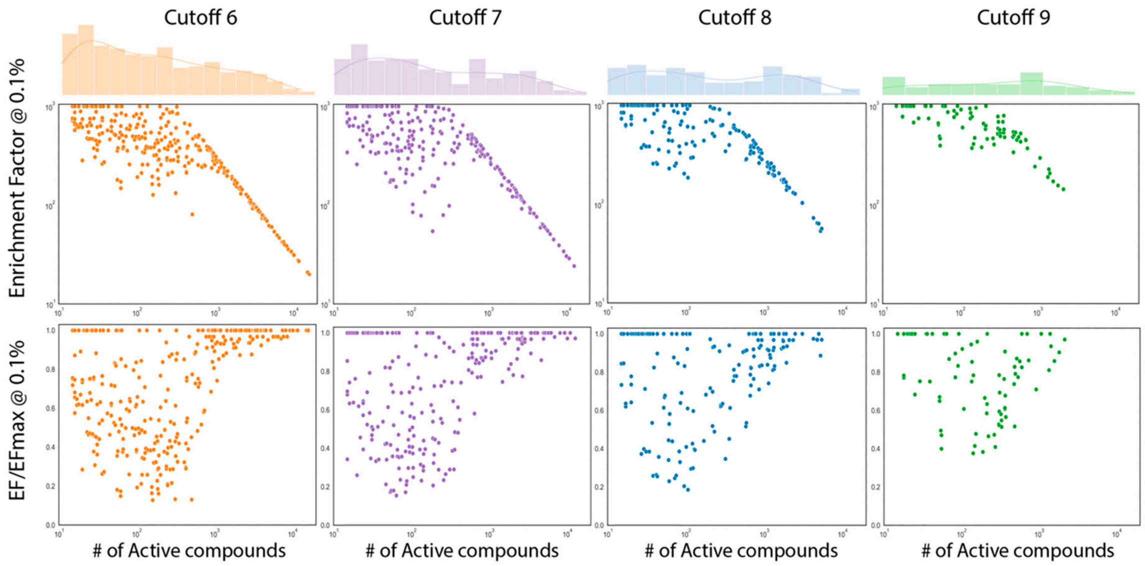


**Figure S11.** Enrichment factor and normalized enrichment factor as a function of active compounds at 0.1% for multitask kinases known active-presumed inactive (KA-PI) classifiers at different pActivity thresholds (cut-off 6, 7, 8, and 9).

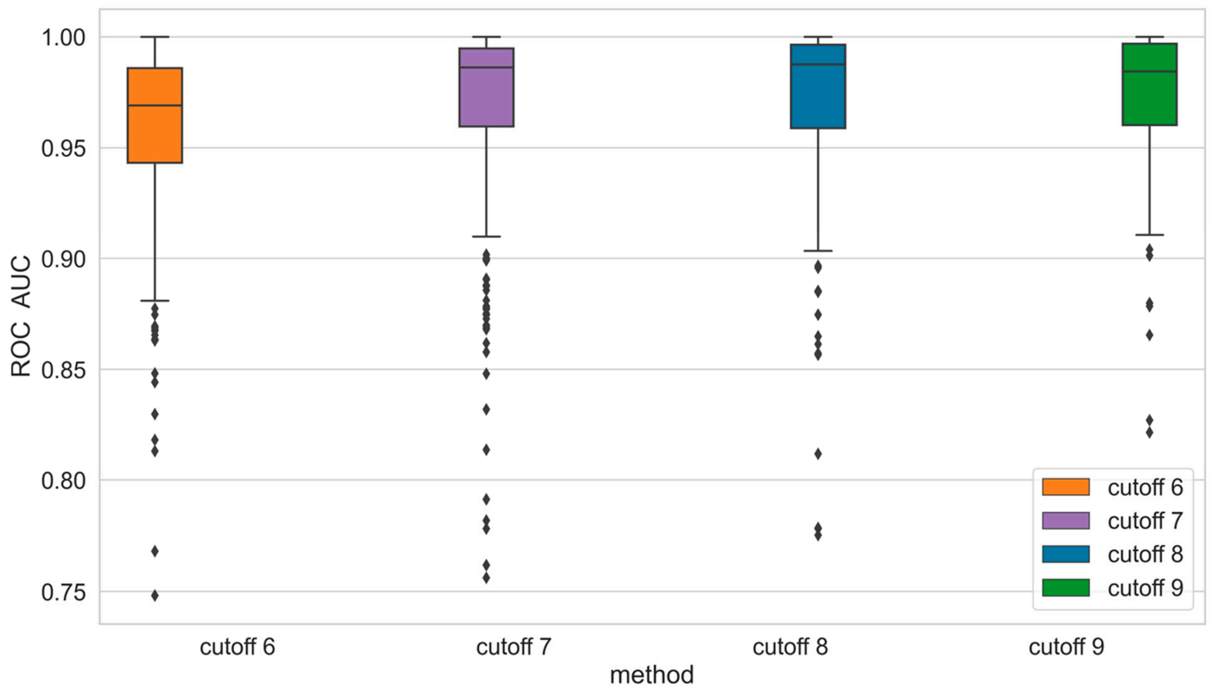


**Figure S12.** ROC AUC comparison for multitask kinases known active-presumed inactive (KA-PI) classifiers at different pActivity thresholds (cut-off 6, 7, 8, and 9).
