# Supplementary material for: Kinome-Wide Virtual Screening by Multi-Task Deep Learning
Source: Int J Mol Sci. 2024 Feb 22;25(5):2538. doi: 10.3390/ijms25052538 (PMC10932040; doi:10.3390/ijms25052538)
Supplement: Supplementary file 1 [file ijms-25-02538-s001.zip › ijms-2800403-supplementary/Document S2/Supporting_Document_LINCS_KINOMEscan_polypharm_figure.pdf]

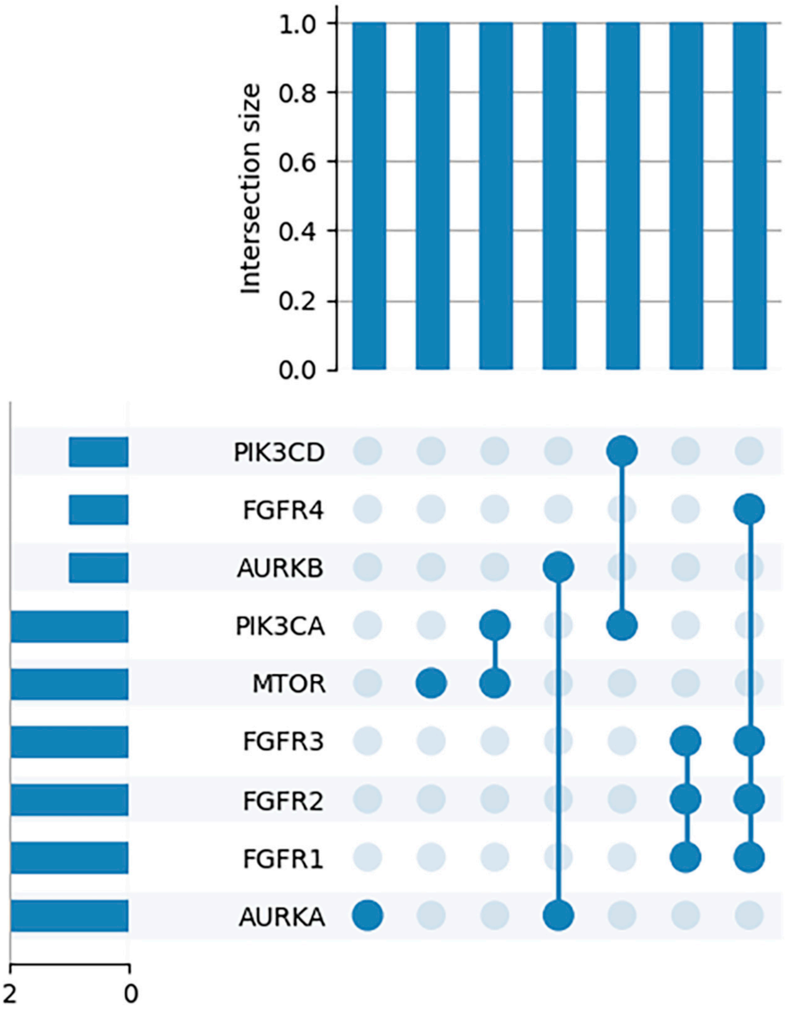

Figure Caption:

UpSet plot summarizes a set of LINCS KINOMEscan compounds displaying polypharmacology activity against the targeted kinases. The bottom left horizontal bar graph of the figure shows the total count of true actives against each kinase target. The top vertical bar graph represents the number of compounds actively against 2 or more kinase targets. Joined blue circles to the right of the targets indicate the unique combination of kinase targets where compounds exhibit polypharmacological effects, providing a visual representation of the overlap in compound activity across different targets.
