# Supplementary figures and images for: Kinome-Wide Virtual Screening by Multi-Task Deep Learning
Source: Int J Mol Sci. 2024 Feb 22;25(5):2538. doi: 10.3390/ijms25052538 (PMC10932040; doi:10.3390/ijms25052538)

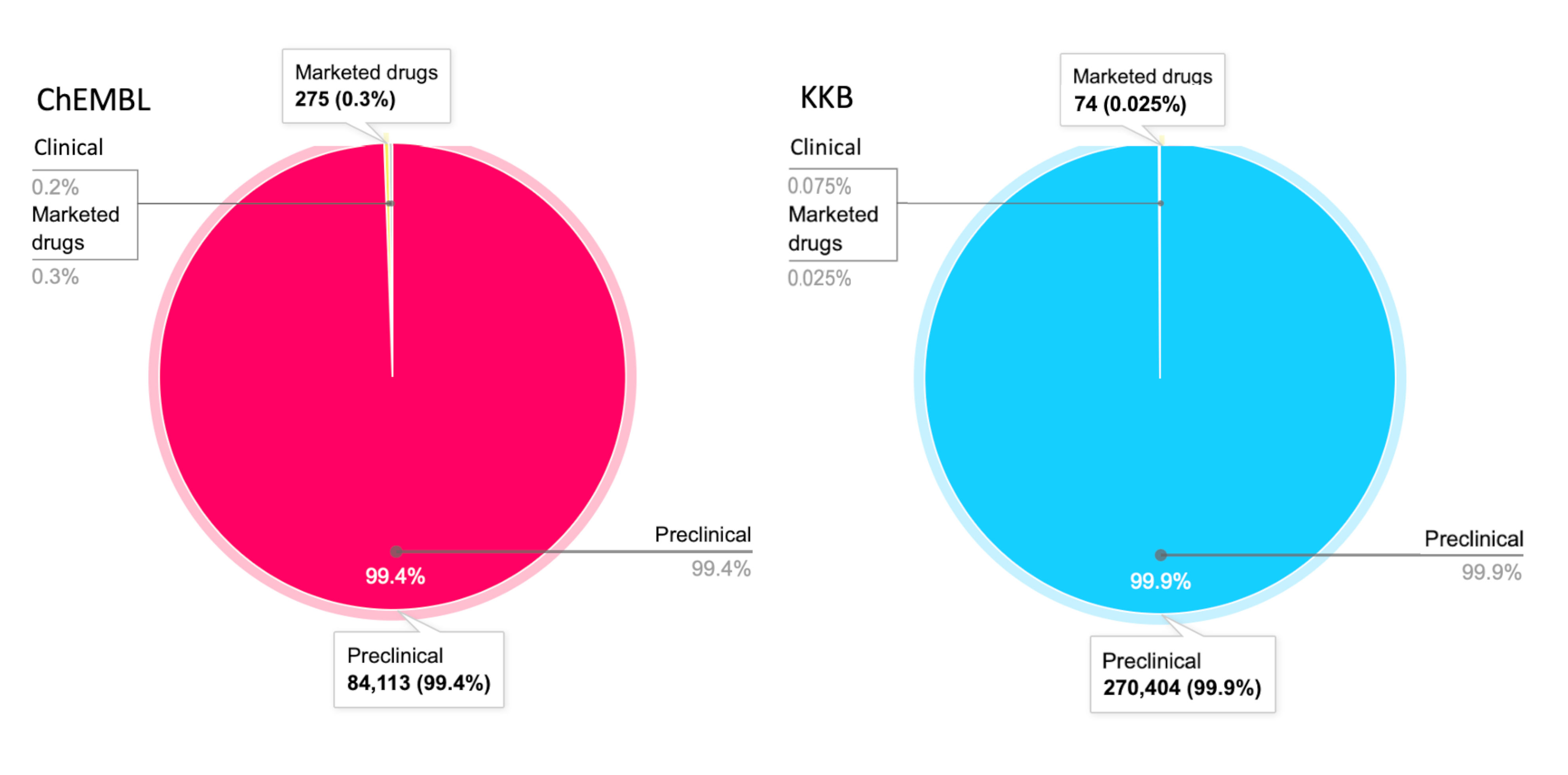

Supplement: Supplementary file 1 [file ijms-25-02538-s001.zip › ijms-2800403-supplementary/Document S1/Figure S1.png]

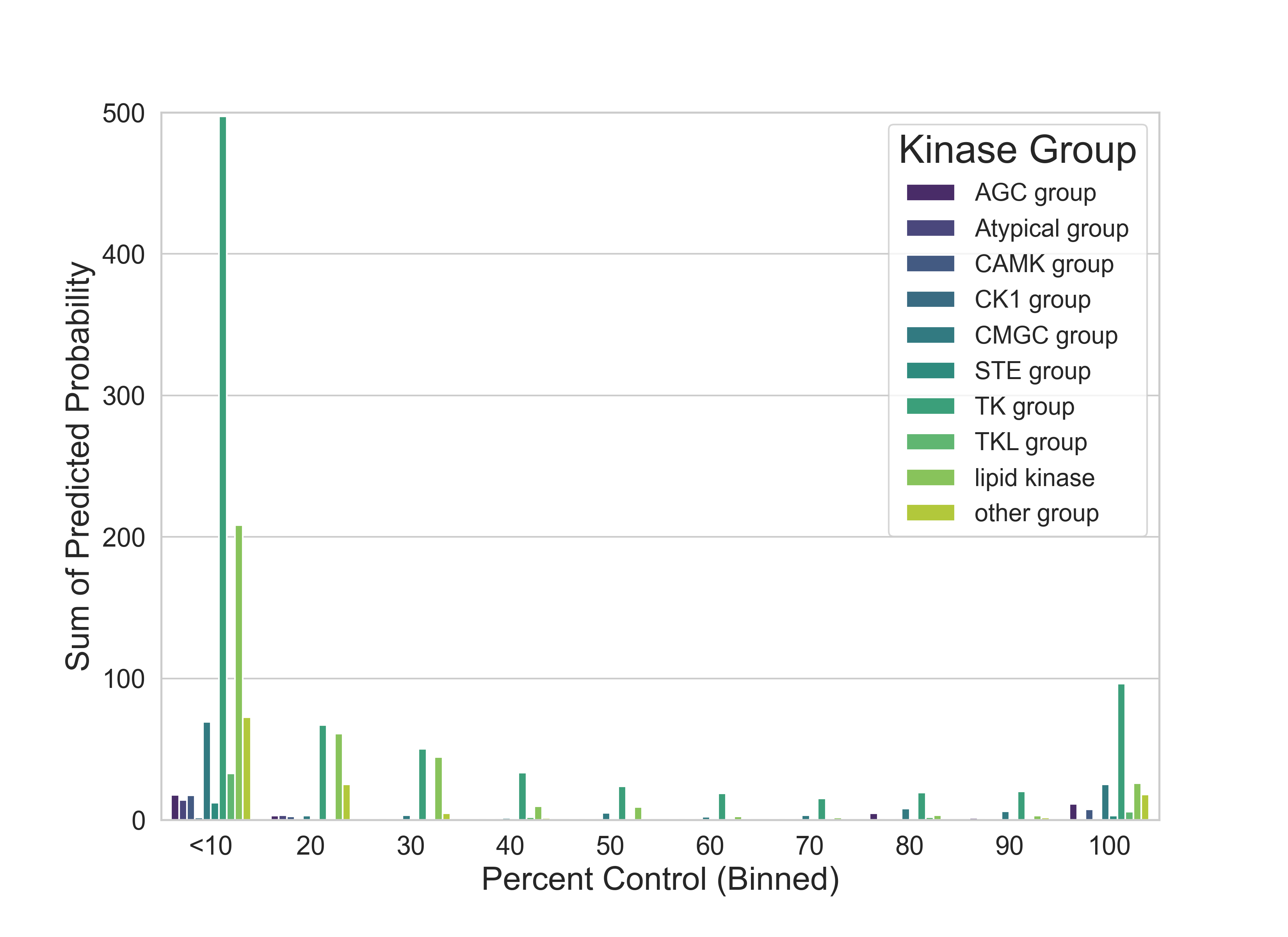

Supplement: Supplementary file 1 [file ijms-25-02538-s001.zip › ijms-2800403-supplementary/Document S1/Figure S10.png]

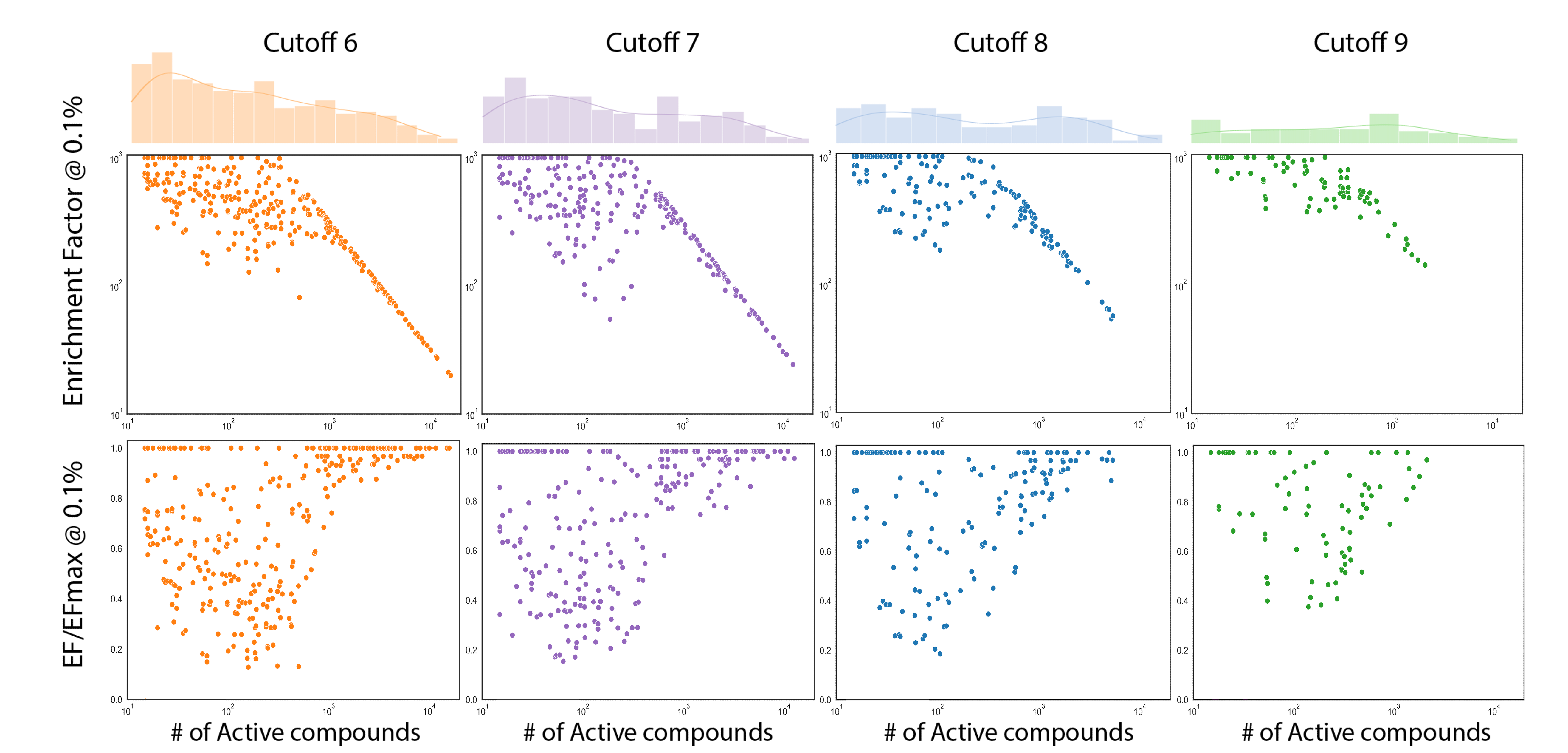

Supplement: Supplementary file 1 [file ijms-25-02538-s001.zip › ijms-2800403-supplementary/Document S1/Figure S11.png]

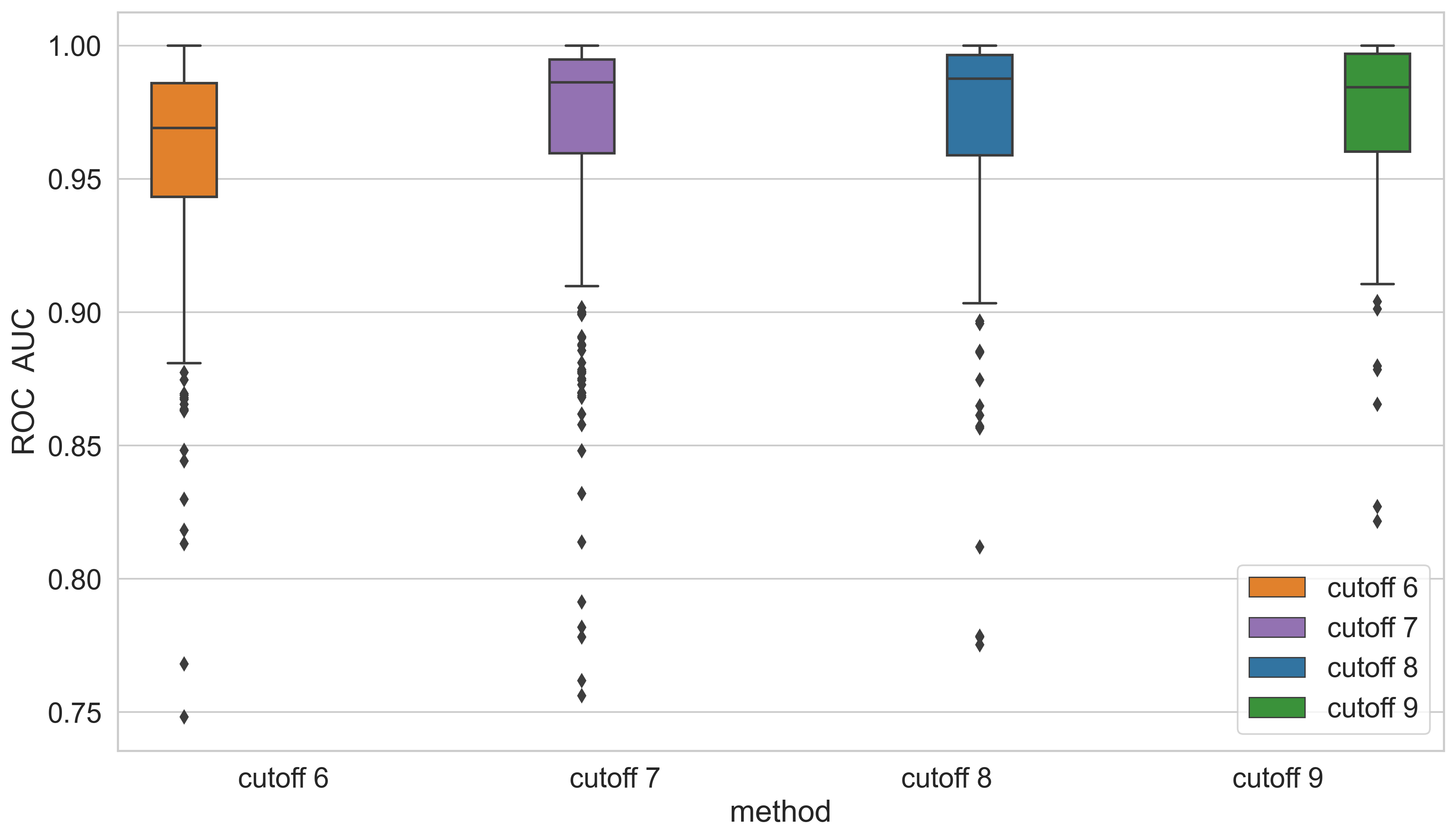

Supplement: Supplementary file 1 [file ijms-25-02538-s001.zip › ijms-2800403-supplementary/Document S1/Figure S12.png]

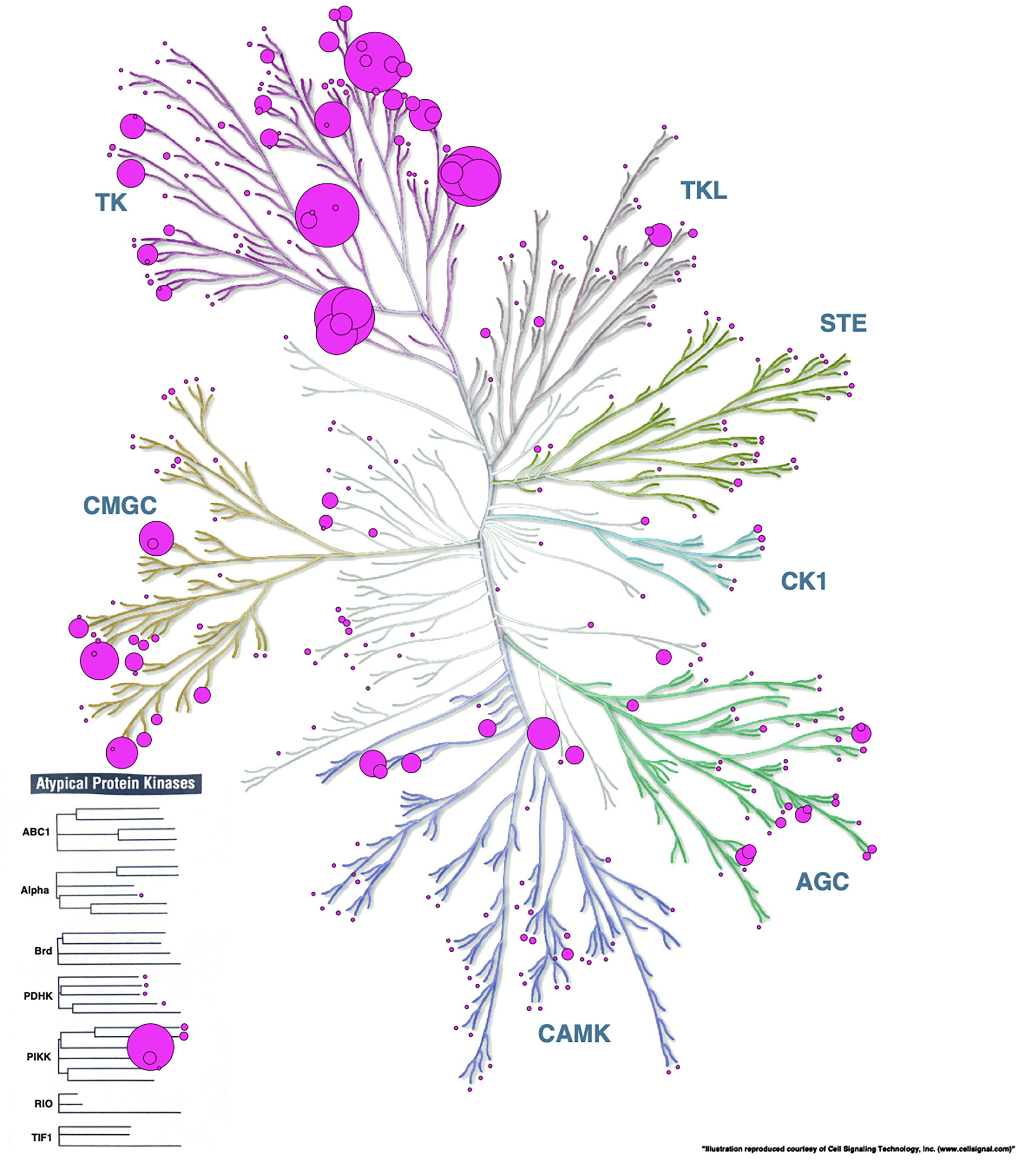

Supplement: Supplementary file 1 [file ijms-25-02538-s001.zip › ijms-2800403-supplementary/Document S1/Figure S2.png]

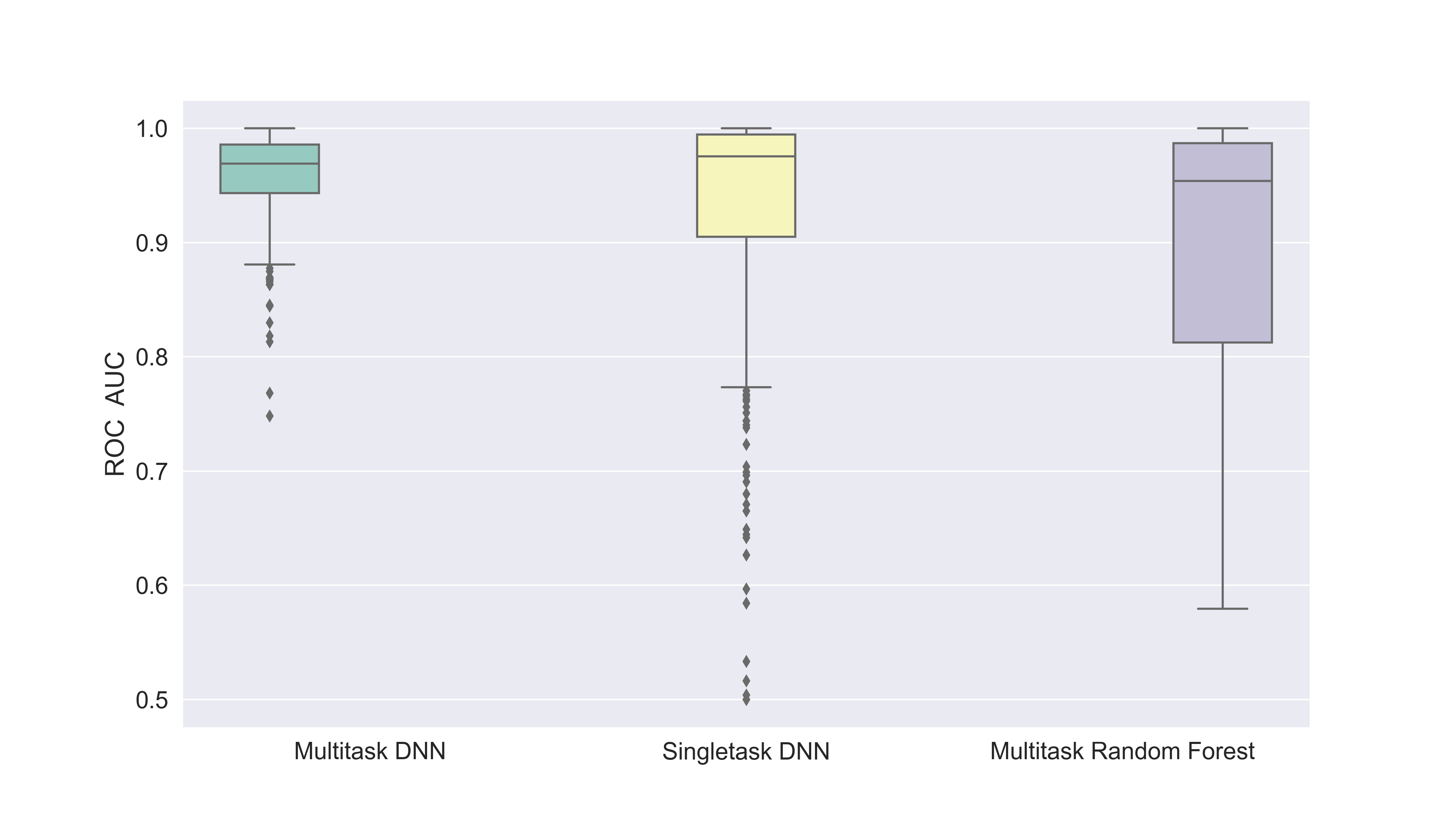

Supplement: Supplementary file 1 [file ijms-25-02538-s001.zip › ijms-2800403-supplementary/Document S1/Figure S3.png]

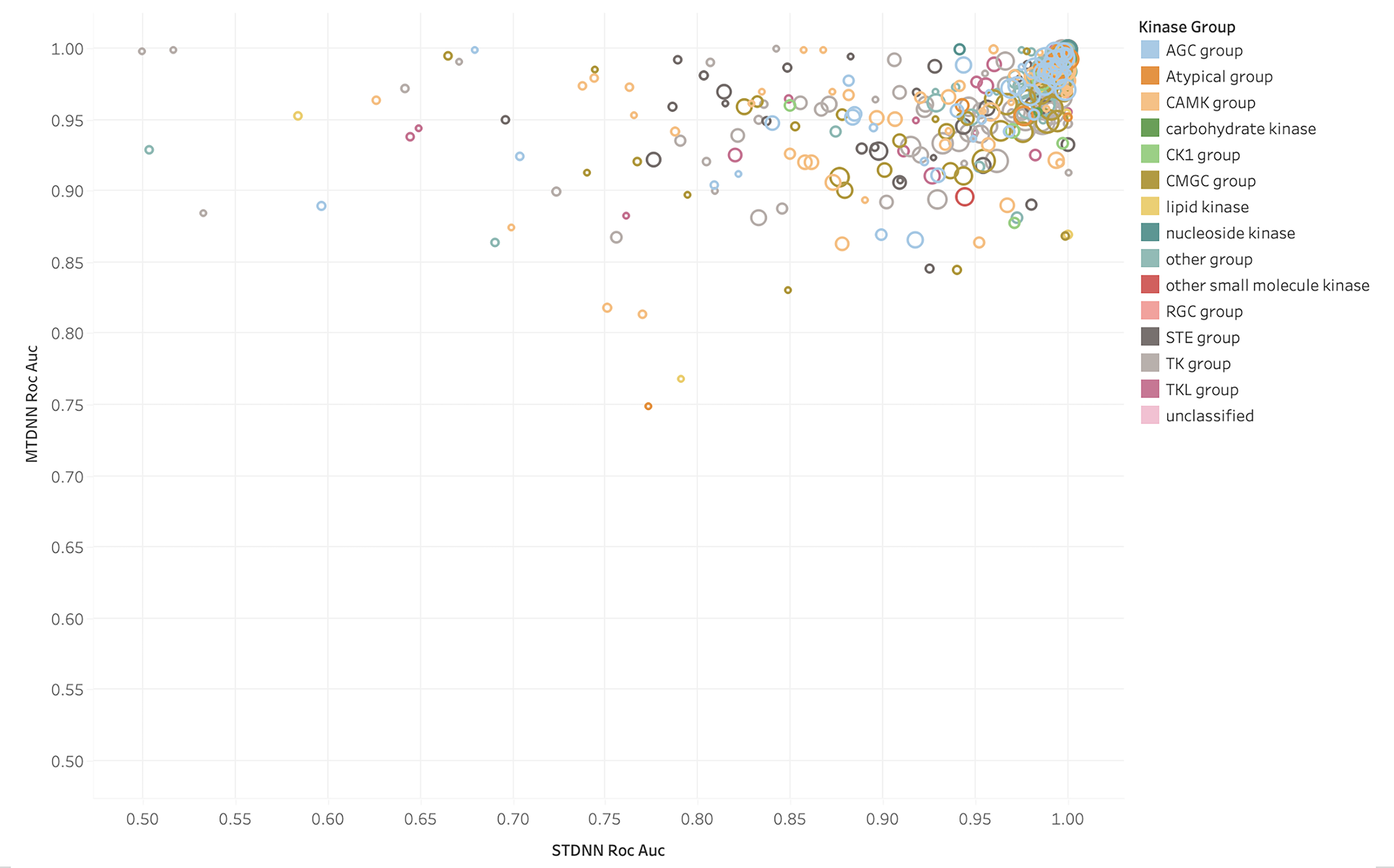

Supplement: Supplementary file 1 [file ijms-25-02538-s001.zip › ijms-2800403-supplementary/Document S1/Figure S4.png]

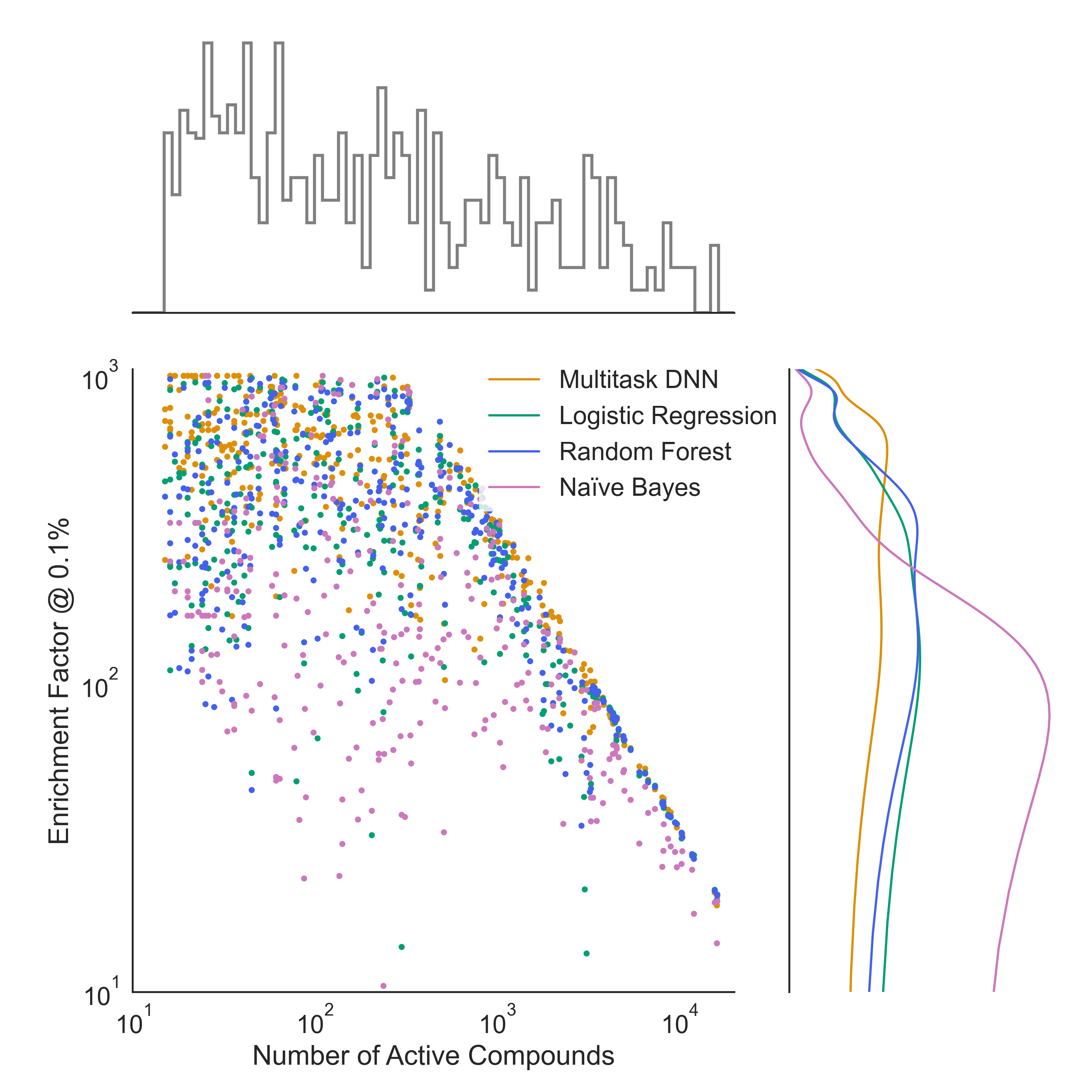

Supplement: Supplementary file 1 [file ijms-25-02538-s001.zip › ijms-2800403-supplementary/Document S1/Figure S5.png]

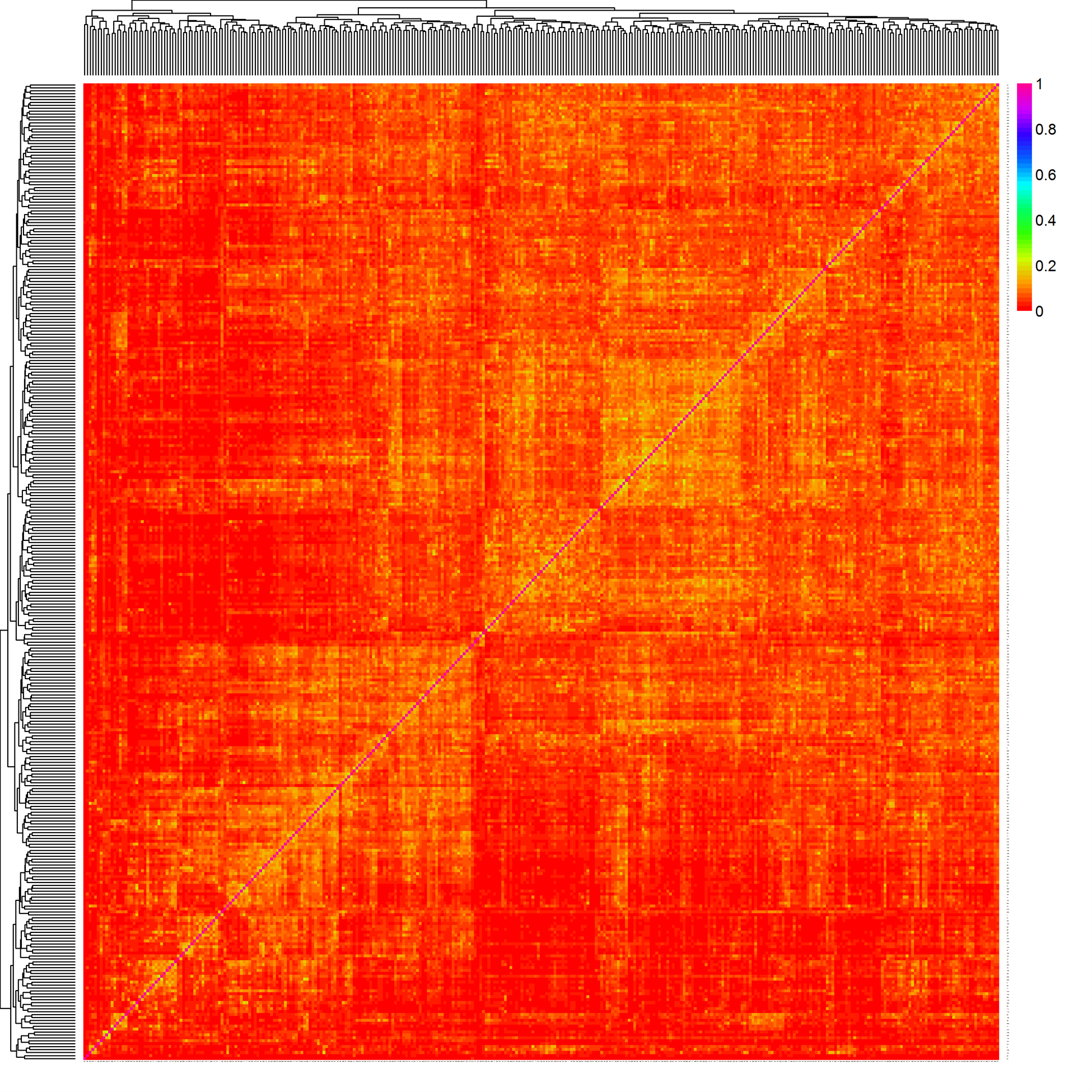

Supplement: Supplementary file 1 [file ijms-25-02538-s001.zip › ijms-2800403-supplementary/Document S1/Figure S6.png]

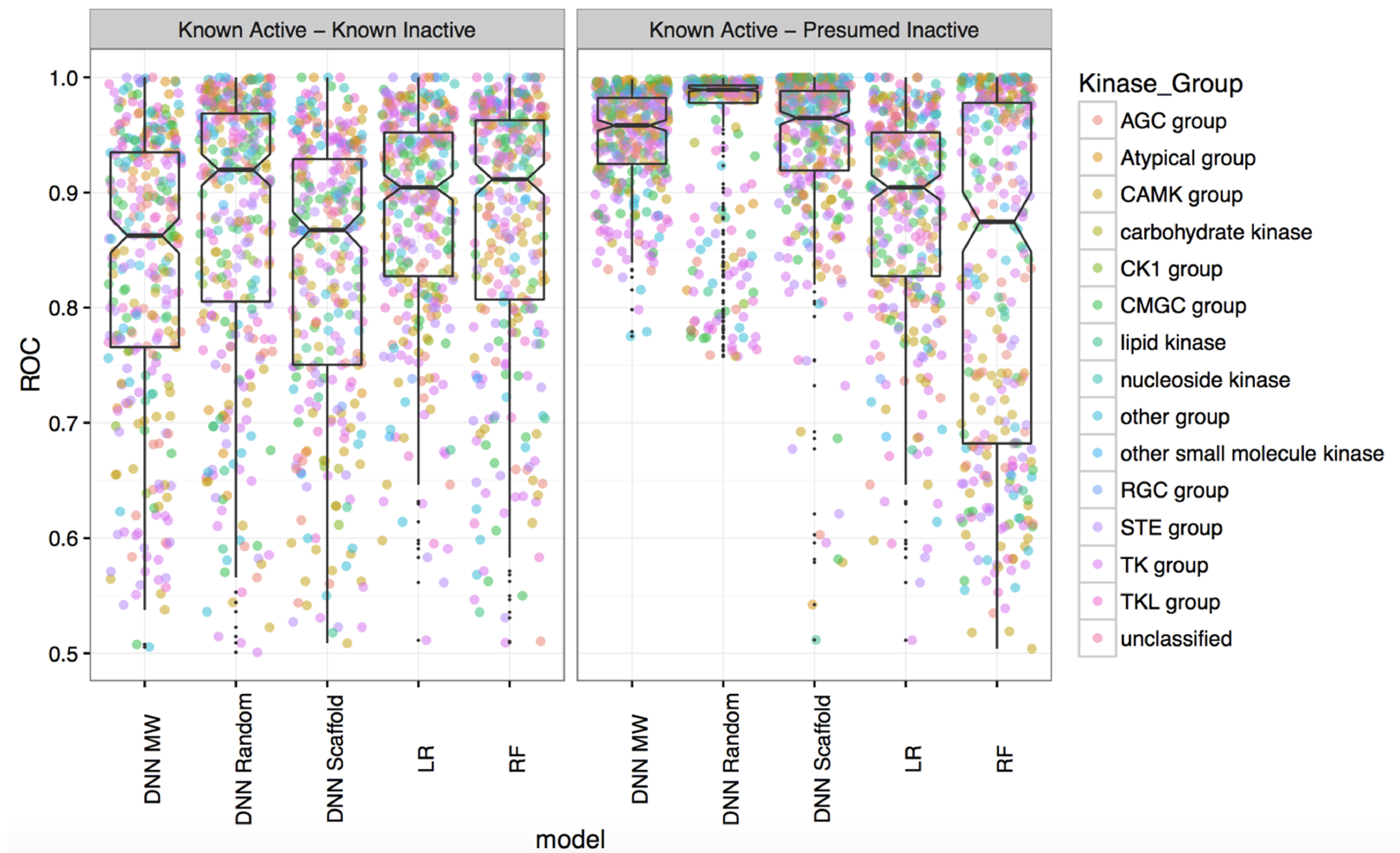

Supplement: Supplementary file 1 [file ijms-25-02538-s001.zip › ijms-2800403-supplementary/Document S1/Figure S7.png]

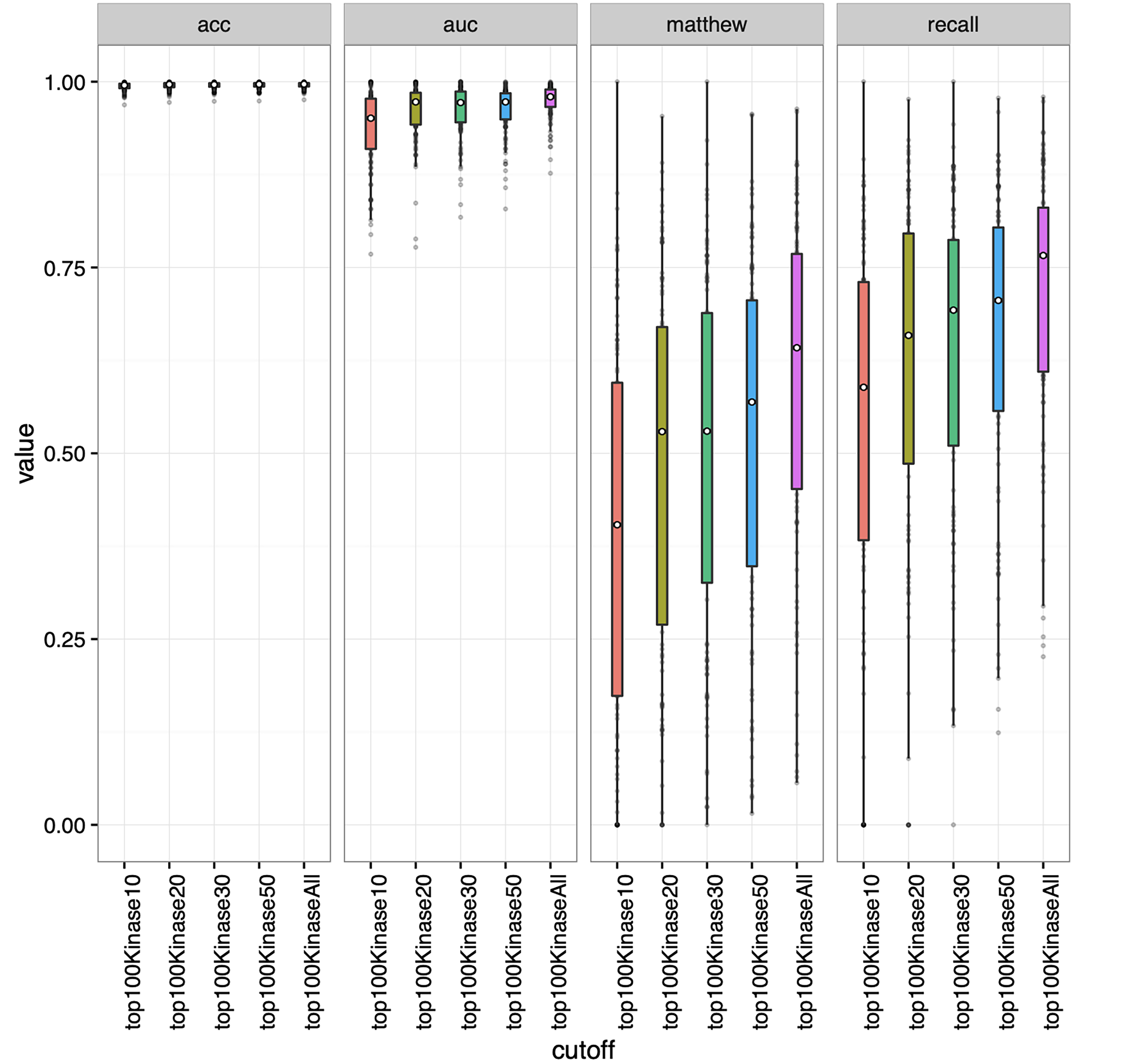

Supplement: Supplementary file 1 [file ijms-25-02538-s001.zip › ijms-2800403-supplementary/Document S1/Figure S8.png]

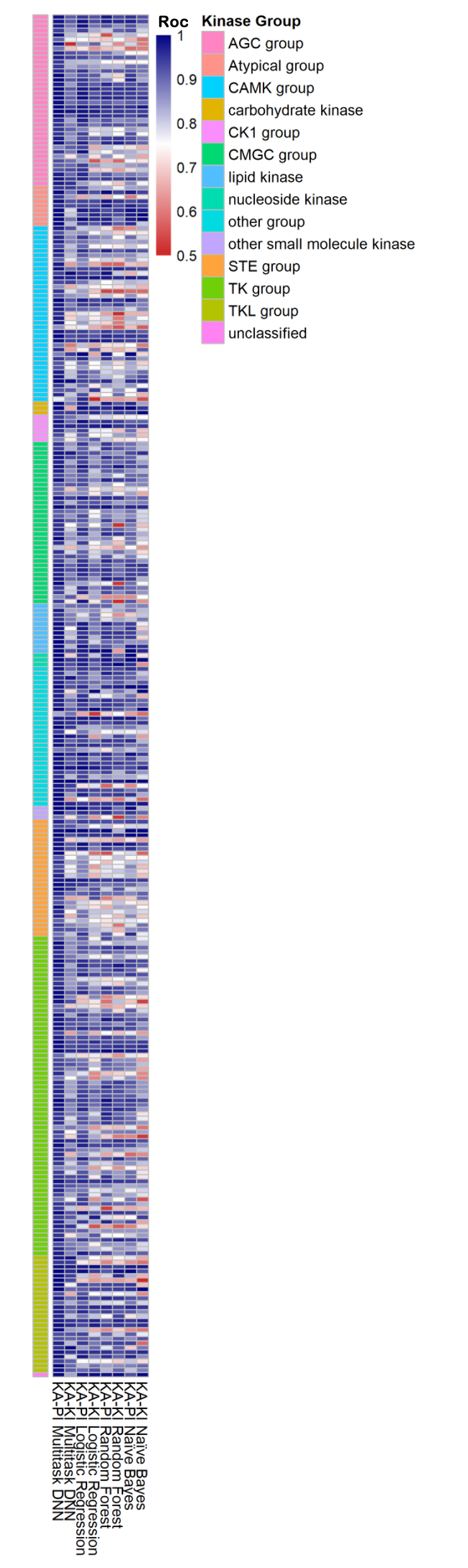

Supplement: Supplementary file 1 [file ijms-25-02538-s001.zip › ijms-2800403-supplementary/Document S1/Figure S9.png]
